# Experiences of caring for large numbers of fallen service members in a realistic large-scale mortuary affairs and emergency burial exercise: maintaining dignity and respect during Aurora 26

**DOI:** 10.3389/fpsyt.2026.1907283

**Published:** 2026-08-28

**Authors:** Jan Grimell

**Affiliations:** Department of Sociology, Faculty of Social Sciences, Umeå University, Umeå, Sweden

**Keywords:** dignity, emergency burial, moral injury, mortuary affairs, triage, war dead

## Abstract

As part of Aurora 26, Sweden’s national total defense exercise involving NATO allies, a large-scale mortuary affairs exercise was conducted. The exercise focused on the recovery, care, and management of large numbers of fallen service members under realistic conditions. With its high level of realism and practical implementation, it represented a scale of mortuary affairs operations not seen in Sweden since the early 1950s. More than 300 military personnel participated, together with several civilian authorities. The aim of this study was to explore military personnel’s experiences of caring for fallen service members, with particular attention to how dignity and respect were maintained throughout the exercise. Data were collected through observations, qualitative questionnaires, and interviews. The material was analyzed inductively, and the use of multiple data sources enabled methodological triangulation. The findings show that participants gained important lessons from the exercise, developed a deeper understanding of the consequences of war, and stressed the importance of training for mortuary affairs operations. At the same time, many described the exercise as emotionally challenging. The operation was carried out by temporarily assembled units rather than a dedicated mortuary affairs capability. Factors that contributed to dignity included ceremonies, the absence of excessive time pressure, a shared commitment to treating the deceased with respect, and adaptive leadership. At the same time, dignity emerged as a context dependent concept that must be balanced against cultural, medical, logistical, and operational demands. Factors that threatened dignity included shortcomings in parts of the logistical chain, fatigue over time, limited training, and problems with administration and documentation. The findings suggest that the benefits of a dedicated, trained, exercised, and properly equipped mortuary affairs unit should be considered from psychological, moral injury, operational, and dignity-related perspectives.

## Introduction

Death is an unavoidable consequence of war. Indeed, it may be argued that death lies at the very core of warfare, insofar as strategy, tactics, military doctrine, technology, weapons systems, materiel, and training ultimately serve the purpose of defeating an adversary through the application of lethal force (1–5). Full-scale wars consistently bear witness to the scale and pervasiveness of death. The ongoing war in Ukraine serves as a stark reminder of this reality, with service members and civilians dying every day on the battlefield, in urban environments, and across civilian communities (6).

The full-scale war in Ukraine has fundamentally altered the conditions of military operations and renewed attention to the realities of death in war (6). Over recent decades, the experiences of many Nordic countries have been shaped primarily by expeditionary, small-scale, and professionalized military operations (7, 8), such as those conducted in Afghanistan and Mali, where the number of fatalities among their own forces remained relatively limited (9). In contrast, the war in Ukraine has reintroduced the prospect of substantial military losses in a high-intensity conflict (6). As a consequence, the ability to recover, identify, and care for large numbers of fallen service members while maintaining dignity and respect has once again emerged as a critical military and societal concern (10, 11).

Despite this reality, death receives comparatively limited attention within military education, training, and exercise activities, particularly when compared to the emphasis placed on other military capabilities. The care of fallen service members, together with the organizational, practical, and ethical challenges associated with large-scale military losses, therefore remains an area that is seldom afforded the same level of attention in military training and exercises as other core military functions. In the event of war, insufficient attention to the recovery, identification, and management of fallen service members, limited training, and inadequately resourced mortuary affairs capabilities become starkly evident once military fatalities become a reality. These deficiencies may pose a significant threat to operational effectiveness, warfighting capability, and overall military readiness (10).

Against this backdrop, and in response to the changing security environment, the Swedish Armed Forces conducted a highly realistic large-scale exercise focused on the care and management of fallen service members within the framework of the national total defense exercise Aurora 26. The exercise involved more than 500 mannequins and body bags representing fallen service members and was conducted on a scale not seen in Sweden since the early 1950s (11). In addition to the Swedish Armed Forces, participating organizations included the Church of Sweden, acting in its capacity as the national burial authority, the Swedish Tax Agency, and the Swedish Defence Conscription and Assessment Agency. Each organization holds specific responsibilities in the processes that follow the death of military personnel. The National Board of Health and Welfare and the Swedish Police Authority were also present to observe the exercise and gather experiences relevant to their respective responsibilities in a scenario of this nature.

In light of the scale and unique character of the exercise, the Swedish Armed Forces’ Chief of Chaplains commissioned a research study to investigate selected dimensions of the exercise through a systematic scientific inquiry. Extensive empirical data were gathered throughout the exercise using participant observation, qualitative questionnaires, and semi-structured interviews. By combining these complementary methods, the study achieved methodological triangulation, strengthening the credibility, validity, and overall robustness of the findings.

### Aim of the study

The study aimed to investigate military personnel’s experiences of caring for fallen service members, focusing on the ways in which dignity and respect were upheld throughout the exercise. Caring for the dead in war is not only a practical and organizational task but also a profoundly moral one, involving questions of responsibility, dignity, respect, and appropriate conduct toward fallen comrades (12–16). Such experiences may have implications for both individual wellbeing and military effectiveness, including warfighting capability (10).

The present article addresses the following research question:

*What experiences emerged from the exercise concerning the care of fallen service members while maintaining dignity and respect?*

This article is the first of two planned publications reporting findings from the study. It focuses specifically on activities involving the Swedish Armed Forces and participating service members. The roles and experiences of representatives from other participating organizations, including government agencies and the Church of Sweden, fall outside the scope of the present article.

The article continues with a review of the relevant literature.

## Mortuary affairs in the military

There is a limited body of peer-reviewed research on mortuary affairs in military contexts. The existing literature has primarily focused on personnel involved in the care and management of the dead from the battlefield to their return home, particularly military mortuary workers within the United States Armed Forces.

In a seminal study, Flynn et al. (13) interviewed 34 military and civilian mortuary workers affiliated with the Joint Mortuary Affairs Center, active-duty Army Mortuary Affairs Companies at Fort Lee, Virginia, and Dover Air Force Base (DAFB) Port Mortuary. Participants described how battlefield deaths occurred under a wide range of circumstances, resulting in human remains that varied considerably in appearance, from individuals who appeared to be peacefully asleep to remains that were severely disfigured, mutilated, decomposed, burned, fragmented, or otherwise altered by the circumstances of death.

The participants reported that wartime environments exposed personnel to intense and often unfamiliar sensory experiences. In addition to encountering human remains, they were confronted with distressing sights, sounds, and smells, as well as environmental stressors such as dirt, toxic exposures, personal danger, and the presence of bystanders. Some remains had been exposed to the elements for extended periods before recovery. Advanced decomposition altered the appearance of the body, including changes in skin coloration, disarticulation of limbs, and extensive insect activity (see also 6, regarding the situation in Ukraine). Furthermore, workers were frequently exposed to large numbers of deceased individuals. Among the sensory experiences described, the odors associated with death and decomposition were reported as the most persistent and psychologically disturbing.

The study found that this work generated substantial occupational stress. Participants employed a range of coping strategies, including emotional distancing, sharing experiences with colleagues, valuing the significance of their work, relying on training and professional experience, seeking support from supervisors and leaders, taking pride in their role, and, when necessary, seeking professional assistance. One aspect of the sharing was using humor. Although to the uninitiated, humor may seem irreverent, callous, and disrespectful. Families were generally not perceived as a primary source of support. As a means of maintaining emotional distance, many workers deliberately avoided learning personal information about the deceased.

Professional support was also sought as a coping mechanism. However, participants reported that mental health professionals often struggled to understand the nature of mortuary work and were not always able to establish credibility or trust. In contrast, military chaplains were frequently identified as the most accessible source of support.

Participants emphasized that, particularly during wartime, mortuary personnel understood the importance of returning the remains of fallen service members to their families as quickly as possible and in the best possible condition. This responsibility contributed to a strong sense of professional pride and purpose. Nevertheless, Flynn et al. (13) described an ambivalent picture characterized by the coexistence of positive and negative experiences. Although workers demonstrated commitment and resilience, they also reported symptoms consistent with traumatic grief and experiences of disenfranchised grief (i.e., grief that is not socially recognized or validated) and were not always able to recognize their own grief reactions. At the same time, despite the considerable emotional burden associated with mortuary work, participants also described meaningful personal growth, a sense of purpose, and other positive outcomes arising from their service.

Several quantitative studies by McCarroll et al. (17–19) consistently demonstrated the psychological burden associated with handling human remains in military settings. Personnel who handled remains during Operation Desert Storm reported significantly higher levels of intrusion and avoidance symptoms than non-exposed personnel (17). Similarly, individuals involved in the recovery and processing of war dead during the Persian Gulf War exhibited significantly higher levels of PTSD symptoms than those who had not participated in such duties. McCarroll et al. (18) further found that personnel who had handled human remains remained at elevated risk for PTSD-related symptoms more than one year after exposure. In a subsequent study, McCarroll et al. (19) reported persistently elevated levels of intrusion and avoidance among personnel who had served in mortuary operations during the Persian Gulf War. Collectively, these findings suggest that service in mortuary operations and exposure to human remains may constitute a substantial psychological stressor, with consequences that can persist long after the exposure itself.

However, McCarroll et al. also identified important individual differences in responses to mortuary duties. Inexperienced personnel reported greater concern about their own emotional reactions and expressed fears of becoming embarrassed in front of others (17). Moreover, both inexperienced personnel and non-volunteers demonstrated significantly higher levels of intrusion and avoidance symptoms than their experienced and volunteer counterparts (17, 18).

Ashbridge et al. (6), in their study of the full-scale war in Ukraine, highlight the challenges posed by environmental conditions and bodily decomposition for the long-term management of war fatalities and the identification of the dead. Their work makes an important contribution to understanding mortuary affairs in large-scale warfare and the complex challenges associated with extensive battlefield casualties. These challenges include the difficulties of recovering and returning the war dead in the face of ongoing threats, as well as situations in which human remains are located in occupied territories or mass graves. Such circumstances make it particularly difficult to uphold the dignity and respect that military organizations, societies, and the families of the deceased seek to preserve in the care and commemoration of the dead. Moreover, the recovery, identification, and documentation of the war dead often continue long after hostilities have ceased, underscoring the enduring human, operational, ethical, and societal responsibilities associated with mortuary affairs in contemporary warfare.

### The moral dimensions of caring for fallen service members

As suggested above, mortuary affairs encompass far more than the technical and administrative management of the dead. They are fundamentally intertwined with ethical and moral considerations that extend beyond the military institution itself and have relevance for society as a whole. Mortuary affairs operate at the intersection of multiple spheres of responsibility, linking the state, the armed forces, the fallen service member, and the bereaved family (13). Consequently, the care of fallen service members cannot be understood solely as a military function, it necessarily reflects and responds to broader societal values, expectations, and moral obligations (15).

A society’s response to its war dead reflects the values, norms, and emotions it seeks to associate with death in war (14, 16). Such responses are not merely legal or administrative in nature. They also embody profound moral considerations concerning what is regarded as right, appropriate, and dignified in the treatment of the dead (12), as well as how best to honor those who have sacrificed their lives in service to their country. The care of fallen service members is therefore often understood as a matter of national honor and collective responsibility, reflecting values that are widely shared across Western societies (13). Furthermore, both society and bereaved families often seek forms of commemoration through which grief can be acknowledged, shared, and alleviated (20).

The expectation that war dead should be treated with dignity and respect is not only embedded in moral norms but is also codified in international conventions and national legislation (21). International humanitarian law, including the Geneva Conventions, establishes obligations regarding the recovery, identification, and respectful treatment of the dead (15). Similar principles are reflected in national legislation governing burial practices and the management of human remains (22). At its core, however, these legal frameworks express a deeper moral commitment, the obligation to do what is right for those who have died in military service. In this sense, fallen service members continue to possess moral significance after death, generating duties that persist beyond the battlefield.

These moral obligations are realized in practice through the processes and institutions that constitute mortuary affairs. They are expressed through adherence to legal and ethical standards, as well as through the recovery, identification, transportation, care, and eventual return of the dead from the battlefield to their families. Mortuary affairs therefore serve not only operational and administrative purposes but also important moral and symbolic functions, embodying society’s commitment to honoring, respecting, and caring for those who have died during military service.

## Mortuary affairs and emergency burial exercise in Aurora 26

The purpose of the exercise was to train the recovery, care, and management of large numbers of fallen service members under realistic conditions. The exercise was conducted as part of Aurora 26, Sweden’s national total defense exercise, which involved NATO allies and took place during late April and early May 2026. However, the exercise involved only personnel from the Swedish Armed Forces and was primarily conducted within the Swedish Navy, which, in the Swedish context, also encompasses amphibious units. The exercise was characterized by a high degree of realism and practical implementation and represented a scale of mortuary affairs operations not seen in Sweden since the early 1950s (11).

More than 300 personnel participated directly in the recovery, care, and management of fallen service members. The personnel involved were drawn from temporary composite units comprising naval and logistics conscripts, regular military personnel, Home Guard units, and personnel from the Central Military Region. At present, the Swedish Armed Forces do not maintain a dedicated unit specifically designated, organized, and trained to conduct mortuary affairs operations.

### Tactical exercise scenario, triage, and declaration of death

Prior to the exercise, Swedish soldiers and seamen had spent several days conducting a range of military tasks as part of Aurora 26, including live-fire combat training. Approximately one week into the exercise, a large-scale mortuary affairs scenario was initiated during the night. Around 100 wounded and lifeless service members (i.e., personnel who had not yet been formally declared dead by a physician) were transported from the archipelago under simulated hostile fire. These casualties were represented by mannequins and weighted military body bags (*bisättningssäckar* in Swedish) intended for wartime use, together with selected items of personal equipment and simulated blood to enhance the realism of the scenario. Combat operations continued simultaneously as wounded and lifeless service members were evacuated from the coastline to a military training area. The exercise then progressed through the medical evacuation chain, including triage, medical assessment, and the formal confirmation and declaration of death by military physicians.

### Activities at the mortuary collection point

At the training area, an additional 500 fallen service members had been positioned at a designated collection point for the deceased, with further fallen service members located at the pier where combat boats arrived to offload wounded and lifeless personnel. The body bags had been filled with materials to approximate the weight of human remains and, in some cases, included items of personal equipment, thereby enhancing the realism of the exercise.

To facilitate the administrative and legal procedures associated with military deaths, the Swedish Tax Agency produced fictitious identities corresponding to the total number of fallen service members represented in the exercise. Each body bag was assigned a unique fictitious identity linked to military identification tags (dog tags), enabling participants to perform all documentation and administrative procedures associated with the confirmation and registration of death as if the fatalities were real. The fallen service members represented Swedish personnel, two NATO allied personnel, and two enemy combatants. Allied personnel and the enemy combatants were included to train documentation procedures and reporting chains.

In addition to the required documentation, all fallen service members were to be treated with dignity and respect throughout the exercise. The transfer of all fallen service members from the collection point at the training area to the Church of Sweden required approximately 13 hours. This period included three commemorative ceremonies led by the commanding officer together with a military chaplain, as well as traditional bugle calls performed by a military musician on two occasions. The process also involved the dignified and respectful carrying, handling, documentation, loading, transportation, and reception of the fallen service members.

### Civil–military cooperation and the conduct of emergency burials

On Church of Sweden property adjacent to the church, participating civilian authorities exercised procedures related to the emergency burial of fallen service members. Both the Swedish Tax Agency and the Swedish Defence Conscription and Assessment Agency developed dedicated exercise systems that enabled participants to practice the administrative processes required following military deaths (also see 11).

The Church of Sweden exercised its statutory responsibilities related to the temporary burial of fallen service members. Although the Church of Sweden was separated from the state in 2000, it continues to perform several public functions. In particular, it remains entrusted by the Swedish state with administering the national burial system in the vast majority of municipalities. In a small number of municipalities, however, this responsibility is instead carried out by other authorities, such as the municipalities of Stockholm and Tranås. This statutory role formed the basis for the Church’s participation in the exercise.

The Church of Sweden conducted what is referred to as a ceremony for the emergency burial of fallen soldiers and seamen. This life-stance-neutral (*livsåskådningsneutral* in Swedish) ceremony emphasized meaning and dignity and was performed in conjunction with the temporary burial procedures exercised during the scenario (11). A life-stance-neutral ceremony is designed to be inclusive and respectful regardless of the deceased’s religion, creed, or personal beliefs. In one sense, it may be described as a secular ceremony, in that it was intentionally free from explicit religious content, while remaining suitable for individuals of all faiths and none. The temporary burial procedures were carried out in full, including the excavation of graves and the burial of fallen service members. It should be emphasized that the exercise did not involve mass graves. Rather, the fallen service members were temporarily interred in individually marked graves, each of which was carefully registered and documented. Such burials are considered temporary, as the fallen service members are intended to be repatriated to their respective home parishes whenever circumstances permit (see also 23).

### Post-incident administrative procedures

The exercise did not conclude once the physical transfer and burial procedures had been completed. For several units within the Swedish Armed Forces, weeks of subsequent administrative work followed, involving casualty reporting and personnel administration related to all fallen service members. Similar follow-on activities were undertaken by the Swedish Defence Conscription and Assessment Agency, the Swedish Tax Agency, and the Church of Sweden. The Swedish Defence Conscription and Assessment Agency also exercised the procedures required for reporting deceased enemy combatants to the National Information Bureau (NIB) in accordance with international humanitarian law, through the International Committee (ICRC) of the Red Cross in Geneva.

## Methods

Given the absence of previous research on this topic in a Swedish context, a qualitative research design was considered most appropriate for addressing the aim of the study. To ensure a broad, robust, and nuanced understanding of participants’ experiences, three complementary methods of data collection were employed.

First, systematic observations were conducted across the entire exercise area throughout the duration of the exercise (24). As observations do not provide direct access to participants’ subjective perceptions and reflections, they were complemented by qualitative questionnaires, which participating service members were invited to complete voluntarily following the exercise (25). Finally, semi-structured interviews were conducted with a small number of participants whose professional roles involved extensive exposure to death and the care of the dead (26). The purpose of the small, purposively selected interview sample was to complement the data obtained from the large number of qualitative questionnaires with more in-depth qualitative accounts informed by professional experience. Drawing on their respective roles as a military physician, military chaplains, and a commanding officer, the interviewees provided profession-specific perspectives that complemented the broader participant data.

Together, the three data sources enabled methodological triangulation, thereby strengthening the credibility and robustness of the findings (27). At the same time, given the nature of qualitative inquiry, findings emerging from a single data source were also regarded as meaningful. For example, observations cannot access participants’ subjective experiences directly, whereas qualitative questionnaires and interviews are specifically designed to explore such perspectives (28).

The research was commissioned by the Swedish Armed Forces’ Chief of Chaplains.

The study was approved by the Swedish Ethical Review Authority (reference number 2026-01258-01).

### Observational study

Systematic observations were conducted, with particular attention paid to how dignity and respect were maintained during the mortuary affairs exercise, as well as to other observable behaviors, interactions, and emotional reactions among participants.

A comprehensive observation protocol was developed by the researcher for this purpose (**Supplementary Appendix 1**). Due to operational and security constraints during the exercise, the observation protocol was provided in paper format rather than electronically.

To ensure adequate coverage of key locations and activities over time, the Swedish Armed Forces assigned 15 observers to support data collection during the exercise. These observers consisted primarily of military chaplains and staff personnel.

Approximately one week before the exercise, the researcher conducted a one-hour online briefing. During this session, the observation protocol was explained in detail, and observers were given the opportunity to ask questions regarding its application. This was complemented by a manual for the observers (**Supplementary Appendix 1**, last page).

During the exercise, the observers were stationed at locations deemed particularly important for data collection and carried out systematic observations. They remained at a distance and did not interfere with the exercise. Upon completion of the exercise, all observation protocols were collected and returned to the researcher for subsequent analysis.

Throughout the results section, findings from the observation protocol are identified by their corresponding protocol number (e.g., Observation Protocol 41).

### Qualitative questionnaire

To obtain a broader and deeper understanding of participants’ subjective experiences during the exercise, participating service members were invited to complete a qualitative questionnaire on a voluntary basis following the exercise.

All participants received information about the study and provided informed consent prior to participation (**Supplementary Appendix 2**).

The questionnaire was intentionally designed to be concise and easy to complete. It consisted of seven overarching questions, one of which contained several sub-questions (**Supplementary Appendix 3**). Due to operational and security constraints associated with conducting research within a military training environment, the questionnaire was administered in paper format and completed manually by participants.

A total of 103 service members completed the questionnaire.

The demographic characteristics of the 103 participants are presented in **Table 1**. according to their respective categories.

**Table 1:** Demographic characteristics.

Category	Participants	Mean age	Average length of service	Gender	Deployment
Conscripts	35	20 years	10 months	29 males, 6 females	None
Full-time serving personnel	34	27 years	5.7 years	27 males, 7 females	5 deployed
Home Guard personnel	27	45.7 years	4.7 years (part-time service)	25 males, 2 females	1 deployed
Part-time serving specialists	7	42,2 years	10,9 years (part-time service)	6 males, 1 female	5 deployed

The conscripts primarily served in amphibious units of the Swedish Navy and in logistics units. They participated in Aurora 2026 as their final field exercise, marking the final phase of their military training.

Among the full-time serving personnel, enlisted seamen and soldiers constituted the overwhelming majority of participants, whereas commissioned officers represented only a small proportion of the sample.

Home Guard personnel serve part-time and are required to complete a specified number of service days annually. While pursuing civilian careers, they retain their military equipment at home, allowing for a high state of readiness and rapid mobilization within their local area of operations.

Part-time serving personnel contributed specialized expertise to the amphibious units in which they served. Owing to the small number of individuals involved, the specific nature of this expertise is not disclosed. In their everyday lives, they pursued civilian careers.

All responses from the qualitative questionnaires were subsequently entered manually by the researcher into a Word document. Participants’ responses were organized by question, with all responses to each individual question compiled into a single document. This process facilitated a comprehensive overview of the dataset and enabled systematic comparison across participants’ responses, although it required considerable manual data management.

Throughout the results section, participants in the qualitative questionnaire are identified as Qualitative Questionnaire Participants (QQP), followed by their participant number and category.

### Interviews

To obtain more in-depth perspectives on experiences related to death and the care of the dead during the exercise, four participants were interviewed following the exercise. Three participants possessed extensive professional experience of working in close proximity to death through their roles as a military physician and military chaplains. The fourth participant was a commanding officer responsible for personnel and operational activities central to the exercise scenario.

All interview participants received information about the study and provided informed consent prior to participation (**Supplementary Appendix 2**).

The semi-structured interview guide was based on themes similar to those included in the qualitative questionnaire, supplemented by profession-specific questions where appropriate (**Supplementary Appendix 4**). The interview format allowed for unplanned follow-up questions and further exploration of emerging topics.

Given the limited number of individuals serving in these specific roles during the exercise, participants are described only at the group level to protect anonymity. Three interviewees, a military physician and two military chaplains, were between 45 and 55 years of age and possessed decades of professional experience. All had been deployed to conflict zones and had substantial experience working in contexts where death was a recurrent feature of professional practice. Both men and women were represented within this group. The commanding officer was in their thirties and had less professional experience than the other interviewees. This perspective was considered particularly valuable given the participant’s responsibility for personnel as well as for operational functions central to mortuary affairs activities.

To further safeguard anonymity, interviewees are referred to throughout the results section as Military Physician, Military Chaplain 1, Military Chaplain 2, and Commanding Officer.

Three interviews were conducted in person on the day following the exercise, while the fourth was conducted by telephone. However, the researcher had met this participant during the exercise itself.

Interview durations were as follows:

- Military Physician: 42 minutes.
- Military Chaplain 1: 64 minutes.
- Military Chaplain 2: 69 minutes.
- Commanding Officer: 50 minutes.

Whereas the military physician and military chaplain 1 primarily acted as observers during the exercise, military chaplain 2 and the commanding officer actively served in their respective roles throughout the exercise.

The interviews were transcribed verbatim by the researcher.

### Conceptualizing dignity in the context of war dead

Although dignity is widely regarded as a fundamental ethical principle, its expression is culturally and contextually shaped. Expectations regarding what constitutes a dignified death and the dignified treatment of the dead vary across societies, institutions, and circumstances (29). This is particularly evident in wartime, where operational constraints, resource limitations, and the realities of armed conflict may challenge practices that would ordinarily be taken for granted in peacetime (6).

Rather than treating dignity as an abstract philosophical property, the present study adopts a practice-oriented conceptualization of dignity in the context of military mortuary affairs. Dignity is understood as something enacted through behaviors, procedures, and rituals that acknowledge the continuing moral worth of the deceased. Such practices include the respectful handling of human remains, careful identification and documentation, appropriate temporary burial procedures, and rituals that honor the dead despite the extraordinary circumstances of war. Within this conceptualization, dignity is not viewed as a static attribute but as something that can be maintained, challenged, or restored through individual behavior, organizational procedures, and institutional rituals.

### Data analysis

Interview transcripts and qualitative questionnaire responses were imported into the qualitative data analysis software Atlas.ti. Given the volume and format of the observation protocols, these materials were analyzed manually rather than imported into Atlas.ti. The analysis of the observation data was conducted alongside the Atlas.ti-based analysis of the interviews and questionnaires to ensure integration of findings across all data sources.

An inductive analytical approach was employed for the interviews, qualitative questionnaires, and observation protocols (30, 31). The inductive approach was particularly well suited to questions concerning experiences during the exercise. It was also appropriate for predefined topics, such as dignity, which were not defined in advance and were therefore open to individual interpretation. Although this may create some tension between a purely inductive and an abductive approach, the analysis presented in this article is described as inductive. It was conducted within the framework of predefined but intentionally undefined concepts that served to guide the exploration without determining the analytical categories.

The analytical process consisted of two main stages: an initial phase of inductive coding followed by a more structured thematic organization of codes.

During the first stage, meaningful units of text were identified and assigned inductive codes grounded in the empirical material. This phase involved detailed, data-driven coding that remained close to participants’ own language and expressions whenever possible. For example, if a participant wrote in the qualitative questionnaire, “An unsettling sight to see churches and tents filled with bodies,” this was coded in Atlas.ti as “unsettling sight of bodies.” The purpose was to remain close to the original accounts while capturing the richness and complexity of the material. In this way, a large number of codes emerged during the initial coding process.

In the second stage, related codes were grouped into broader subthemes in Atlas.ti. This process facilitated the identification of recurring patterns and subthemes across the dataset. For example, the code “unsettling sight of bodies” was grouped under the subtheme “Strong visual impression,” which captured different accounts describing the visual impact of body bags. While the transition from detailed, lower-level codes to subthemes inevitably involved some loss of nuance, it was necessary to achieve a coherent, analytically meaningful, and transparent presentation of the findings.

As a final step, findings from the interviews, questionnaires, and observation protocols were compared and triangulated. Subthemes identified across all three data sources were considered particularly robust because they reflected convergence between multiple forms of qualitative evidence (27). At the same time, subthemes emerging from only one or two sources were not discounted, as different methods provide access to different dimensions of human experience. For example, participants’ subjective experiences and reflections could not be captured through observation alone and therefore appeared primarily in questionnaire and interview data. Consistent with qualitative research principles, such findings were nevertheless considered important and valid contributions to understanding the phenomenon under study (28). These findings are therefore also included in the presentation of results.

Finally, overarching themes were developed to organize the subthemes into a higher-order analytical structure. For example, subthemes relating to experiences during the exercise were organized under the overarching theme “Experiential impact.” The analysis generated three overarching themes, each encompassing a number of subthemes, as presented in **Table 2**.

**Table 2:** Overview of overarching themes and subthemes identified in the analysis.

Overarching themes	Subthemes	Codes
Experiential impact	Deeper insights and understanding of death and war Strong visual impression Emotionally affecting Important to train because “we had not been thinking war” Pride	Numerous individual codes
Dignified treatment	Ceremonies and the cultural perspective Absence of stress The medical perspective The logistical perspective	Numerous individual codes
Threats to human dignity	The entire logistical chain must function with dignity The time factor Documentation	Numerous individual codes

In the Results section, the analysis is presented through the overarching themes and subthemes, which serve as headings and are illustrated with excerpts from the empirical material to ensure transparency throughout the analytical process. To protect participant confidentiality, expressions, contextual details, and other information that could directly or indirectly identify individual participants have been rephrased, anonymized, or omitted.

### Rigor and trustworthiness

The study was approved by the Swedish Ethical Review Authority and conducted in accordance with established principles of qualitative research. Data from interviews, qualitative questionnaires, and observation protocols were analyzed using an inductive analytical approach, allowing patterns, themes, and interpretations to emerge from the empirical material while generating new insights into the phenomenon under study (30, 31). The analysis was conducted systematically through repeated engagement with the data, inductive coding, thematic categorization, and iterative refinement of emerging themes (26).

Credibility was enhanced through methodological triangulation across the three data sources (27). Convergence across interviews, questionnaires, and observations was regarded as an important indicator of analytical robustness, while findings unique to a single source were retained when they illuminated dimensions of experience inaccessible through other methods. Trustworthiness was further strengthened through continuous comparison between codes and themes, as well as through the presentation of rich empirical material in the results section, enabling readers to assess the relationship between the data and the interpretations presented (26).

The translation of Swedish-language accounts into English was undertaken iteratively by the researcher, moving back and forth between the original text and the translation to preserve both meaning and contextual nuances. The original Swedish-language material is available from the corresponding author upon reasonable request.

The study was conducted through Umeå University, where the researcher is employed as a Senior Lecturer, thereby ensuring academic independence and scientific integrity despite being commissioned by the Swedish Armed Forces. The researcher’s professional background as both a military officer and an ordained priest provided an in-depth understanding of the organizational culture and operational context. At the same time, reflexivity was maintained throughout the research process through continuous critical reflection on how the researcher’s professional background and contextual proximity to the field could influence data collection, interpretation, and analysis. These considerations informed ongoing efforts to critically examine assumptions and minimize potential bias.

## Experiential impact

### Deeper insights and understanding of death and war

A recurring theme in the collected material was that the exercise had provided deeper insights into both death and war. For most conscripts, as well as many officers, death had largely been an abstraction, something discussed in theory, but rarely approached directly or practiced in any meaningful way. Notable exceptions included more advanced medical units, military chaplains, and the small number of participants with prior international deployments who had, in one way or another, prepared for the possibility of their own death.

The mortuary affairs exercise therefore contributed to a deeper understanding of the military profession and of a self-evident yet rarely rehearsed consequence of armed conflict: that comrades, and oneself, may die. Rather than having a negative effect on participants, this appeared to foster a form of reflective maturity.

“I was also confronted with the realization of how close death is for all of us and gained a clearer understanding of the consequences of war.” QQP 4

“I had never thought about how many resources it takes to move the fallen from the point of contact all the way back to burial.” QQP 14

“It is a difficult task that everyone should practice. It is very important to understand what these duties involve.” QQP 15

“It is difficult to imagine the emotions I would have experienced in a real situation, as it is unlike anything else I have encountered in life. I believe the task would have been far more affected by psychological strain if it had been real, which would have made it harder to carry out.” QQP 20

“The day raised many thoughts. For example, that this is constantly happening around the world. Also how long the whole process takes, which it should.” QQP 28

“That war and death are a terrible reality in the world, and that it is important to handle that reality with dignity and respect, for our comrades and for the families of the deceased.” QQP 43

“It is the hard truth about what can happen and what must be done.” QQP 59

“It is necessary to understand that this could become a reality, so that we are prepared if the worst happens. We cannot look away; instead, we must face the really difficult things with wisdom, understanding, respect, and dignity.” QQP 79

“How demanding a similar task would be in real life.” QQP 81

“Good to go through, it is talked about too little, and it is important to actually practice it!” QQP 97

The participants’ deeper reflections on death and war also brought to mind the situation in Ukraine, where death on the battlefield remains a daily reality for service members.

### Strong visual impression

In addition to the evacuation of around 100 casualties in the archipelago, 500 filled body bags had been placed at a collection point for the fallen, located some distance behind the triage area and medical units. The sight of 500 body bags lined up in rows was something participants repeatedly returned to.

“Seeing that many bodies lined up is insane. It is really something you never want to see for real.” QQP 24

“Completely macabre, seeing the body bags.” QQP 49

“It was a shocking sight to see so many lying there in rows.” QQP 96

“When we arrived at the site, it looked and felt very macabre.” QQP 97

“The first time I came down to the quay and saw them all, when the sheer number of fallen becomes visible, it really sets a tone of seriousness. Out on the pier there were maybe 70 lined up. It becomes very visual [ … ] you understand intellectually that people die in war, but it remains an abstract idea, and this made it very concrete.” Military Chaplain 1.

The visual impression was powerful. The large number of body bags removed any sense of abstraction and made the ultimate consequences of war tangible and immediate, a finding also noted in Observation Protocol 1.

### Emotionally affecting

All participants reported that they were generally doing well after the exercise, although some described feeling tired. At the same time, many participants reported having been emotionally affected by the exercise in different ways.

“The thought that this could happen to my closest comrades is a heavy one.” QQP 10

“I was more affected than I expected to be, even though they were not real bodies.” QQP 18

“It felt difficult to imagine that it could actually have been your own comrades.” QQP 21

“There were also feelings of sadness and gloom.” QQP 36

“It affected me emotionally more than I thought it would.” QQP 54

“I feel that the exercise was tough psychologically because it made me realize that this could happen here one day, and that it happens elsewhere every day.” QQP 75

“It is extremely challenging mentally, physically, and emotionally. Emotionally, the exercise puts into perspective what we actually work with. Comrades will die.” QQP 93

“Hard to see so many dead.” QQP 94

“It is moving when you think about all those body bags being filled with very young adults, the same age as my child. Seeing all the body bags while we were also transporting casualty role players back and forth was affecting.” QQP 101

“One thing I reflected on is that death is quite abstract for many people. Many have never seen a dead person. [ … ] It prompted a lot of reflection among people passing by, and many came up and spoke about it spontaneously. People automatically lowered their voices when walking past, and when they moored at the pier where there were large numbers of body bags, some became visibly emotional. Many said that “it hit hard” to see it all.” Military Physician.

Participants were clearly affected emotionally in different ways, often in relation to comrades and family members. The importance of military cohesion within units and teams, as well as the relationship between battle buddies, is well documented in the research literature. However, the deeper emotional consequences of losing comrades cannot be simulated through training.

### Important to train because “we had not been thinking war”

A prominent theme in the collected material was the realization of how important it is to train mortuary affairs in order for all parts of the process, from triage and pronouncement of death to the dignified handling of the fallen throughout the chain of care, to function effectively in a real situation. Participants expressed the view that the armed forces had not truly been “thinking war” in terms of large scale loss of life. There was a strong consensus that this should be as integral to military education and training as any other military activity. Participants emphasized that effective, dignified, and respectful handling of the dead requires extensive training at multiple levels. It is not something that happens automatically. Moreover, it involves both military and civilian actors, adding further complexity.

“We have never really been thinking war in the sense that this would be needed. War has largely been something that happened abroad, where we planned for perhaps one fatality and two wounded at most. Our resources were organized accordingly, allowing us to provide very high quality care in those situations. [ … ] From a medical perspective, this will require a major adjustment. One thing people rarely talk about is having to refrain from doing things that could be done. I think that will be a major challenge, as will witnessing people die without being able to intervene.” Military Physician.

“This exercise is very important. This is something the Swedish Armed Forces must be able to handle in a good and dignified way. Many parts need to work, and many different actors need to do their part.” QQP 10

“I think it is a bit crazy that this is not practiced more often. It is still a major part of warfare.” QQP 11

“I am surprised it has been 72 years since this was last practiced. This is at least as important as everything else we train for. The Swedish Armed Forces need to train this more.” QQP 12

“Training for this is a success factor for the Swedish Armed Forces.” QQP 13

“I also feel that we still have a long way to go before we can take care of our dead in the way they deserve.” QQP 20

“I think exercises of this kind, dealing with activities that take place around the actual fighting, are extremely valuable and deserve more attention. They also improve coordination between the Swedish Armed Forces and other authorities, which is crucial in times of crisis and war.” QQP 87

Another finding related to the exercise was its pedagogical and reassuring effect on participants themselves in the event that they were to become casualties. This appeared to be linked to having witnessed and actively participated in the mortuary affairs exercise.

### Pride

Another theme identified in the material was the participants’ subjective experience of feeling proud, honored, and grateful to have taken part in the exercise and contributed to the mission involving the fallen.

“I feel proud of the part of the exercise that my group and platoon took part in. We did a very good job and maintained dignified handling of the dead all the way through.” QQP 3

“It was an honor to participate in such a large operation, and the logistics worked very well.” QQP 4

“I feel grateful for the trust placed in me and that I contributed to an important task.” QQP 5

“I felt that the exercise was handled in a dignified way throughout, which made me proud to take part.” QQP 18

“Looking back now, I feel proud.” QQP 28

“Emotionally, feelings of pride dominate, both in my soldiers and in my own performance, together with satisfaction at having completed the task and inspiration from meeting new colleagues who were equally focused, professional, and kind.” QQP 36

“Proud to have taken part.” QQP 74

“Pride. Dignity. Motivating.” QQP 77

“So grateful that we managed to carry out something so important. Grateful for the commitment and efforts of everyone involved.” QQP 79

“I am proud to have been part of this. The last time this was practiced was, I believe, in 1952, so I am proud to have contributed.” Commanding Officer

The underlying reasons for these emotional responses are not easily disentangled. One possible explanation is that an exercise of this scale had not been conducted for a very long time, which may have fostered a sense of successful pioneering. The task was carried out with a high degree of realism, and participants successfully solved it together. At the same time, the mortuary affairs mission, particularly in the context of mass casualties, recovery operations, triage, pronouncements of death, large numbers of fallen personnel, and emergency burials, was framed by a profound sense of seriousness and urgency. These realities directly concerned the participants and their comrades, creating a distinctive military context for these emotional experiences.

## Dignified treatment

### Ceremonies and the cultural perspective

An important aspect of mortuary affairs, given legal requirements and established conventions, is the maintenance of dignity and respect for the dead. This is further reinforced by the fact that mortuary affairs concerns service members who have sacrificed their lives for their country and whose deaths are associated with strong emotional bonds among comrades and family members. This also extends to allies with whom one has fought side by side, as well as enemy combatants.

What constitutes dignified and respectful treatment can be understood as culturally situated and defined by the culture in which it takes place. It is also dependent on context and circumstance. In the Swedish context, dignity and respect are shaped both by broader societal understandings of death and by military culture.

In Sweden, where the Church of Sweden is generally responsible for burial services, practices and rituals surrounding death have strong historical and contemporary connections to how churches and clergy create dignity around death and burial. Clergy possess extensive experience of death and are experts in how death is managed within a Swedish cultural context. Clergy also serve as military chaplains within the Swedish Armed Forces.

As part of the mortuary affairs exercise, three memorial ceremonies were conducted at different points during the day. These were led by the commanding officer and a military chaplain, with a musician participating on two occasions. The ceremonies combined elements associated with military dignity, such as removing berets, standing at attention, and saluting, with broader ritual practices surrounding death represented by the chaplain in a nonconfessional and inclusive manner.

Participants experienced these ceremonies as meaningful ways of honoring the fallen and creating dignity and respect, a finding also documented in numerous Observation Protocols (e.g., 1, 6, 14, 18, 22, 25, 37, 49, 83).

“Service members saluted as we passed. The formations and ceremonies honoring the fallen felt dignified.” QQP 10

“All the ceremonies that were held were very moving and meaningful.” QQP 11

“I thought it was fantastic that ceremonies were held for the fallen.” QQP 14

“Of course, as a comrade or family member, you would want the funeral or ceremony to be more personal, for example mentioning the person’s name, but that becomes difficult when there are so many deceased.” QQP 43

“Powerful emotions throughout the entire ceremony.” QQP 55

In this context, the military chaplain emerged as a key figure with an important role. Rituals surrounding death fall within the chaplain’s area of expertise, whereas they are not part of the officer’s professional domain. The integration of ecclesiastical expertise regarding ceremony and ritual with military practices created a sense of cultural dignity that also provided support to participants. One participant who was unable to attend the ceremony expressed it as follows.

“I did not take part in the ceremony and only helped load the bodies, which made everything feel very task focused and I received no explanation or support. Everyone should have the time and opportunity to take part in the ceremony and receive support. I think that is important.” QQP 21

The ceremonial dimension may also be understood as a cultural perspective concerning how the dead should be surrounded by rituals in order to be treated with dignity and respect. Closely connected to this is the compassionate care of the grief and emotions experienced by surviving service members. Within the Swedish military context, these aspects are addressed through the framework of military soul care, a responsibility carried out by military chaplains. In this sense, rituals serve not only to honor and care for the dead, but also to support the living. The dimension of grief itself was difficult, if not impossible, to simulate within the exercise.

### Absence of stress

Military activities are often associated with stress, time pressure, rapid decision making, and tasks that must be completed within strict deadlines. In short, military work is typically expected to be fast and efficient.

A clear theme in the collected material was that the deliberate absence of stress and the explicit commitment to carrying out the mission, namely caring for and transporting the fallen, in a dignified manner was central to participants’ experience of dignity. This was also noted in the majority of Observation Protocols. The absence of time pressure, deadlines, and officers urging personnel to increase the pace stood in marked contrast to ordinary military activities. Mortuary affairs was therefore experienced as something different, closely linked to leadership practices.

“My group maintained the same standard throughout the exercise, while others became stressed and lost some of the dignity in how they handled the dead.” QQP 11

“During loading and unloading, I felt that my comrades and I handled everything with dignity, from carrying the bodies to securing them during transport. We even drove more carefully.” QQP 14

“We loaded the fallen with dignity and were genuinely careful and respectful.” QQP 15

“It was better for things to take a little longer and be dignified than to be fast and efficient.” QQP 20

“I thought the bodies were treated with respect and dignity. This was shown by being careful and maintaining a calm and controlled pace.” QQP 24

“Throughout the day and the entire process, everything was done calmly and methodically. There was never any pressure or stress.” QQP 28

“I felt that dignity was maintained and actively pursued. A major reason was the absence of stress. Stress is often what creates a difficult atmosphere. In most exercises officers are pushing people, saying things like, “increase the pace” or “you have seven minutes for this.” There are many lessons to learn from doing things properly and without stress. It was maintained because everyone had decided that it should take the time it needed and that it should be done correctly. That made it very respectful. [ … ] Everyone involved, especially those leading the activity, seemed committed to making dignity a priority.” Military Chaplain 2

One way in which dignity was operationalized during the exercise was therefore through deliberately reducing the pace and avoiding unnecessary stress. This involved carrying the fallen in a controlled manner, moving at an appropriate pace, and avoiding rushing processes such as loading and unloading. This approach reflected the explicit intention of leaders to allow activities to take the time required in order to preserve dignity and prevent the fallen from being treated in a hurried manner.

A central element of leadership was the clear message that the work should not be rushed. By giving personnel sufficient time to complete their tasks without pressure, the principle of dignified and respectful treatment of the fallen was translated into practice.

This perspective may also be understood as a cultural one in the sense that behaviors and practices that would normally be standard procedure were deliberately modified in order to foreground dignity and respect.

### The medical perspective

The collected material also revealed a medical perspective. This reflected a somewhat different understanding than the cultural perspective regarding what constituted dignified treatment of the fallen.

“I think dignity lies in being cared for, being moved away, and being placed in a grave rather than being left where you are. In that sense, efficiency itself can be more dignified and more dignity creating. [ … ] If all these people had died during the night, there would probably not have been much smell or fluid. After death there is not much bleeding, so there would not have been large amounts of blood in the bags. Maybe a little, but not much. Other fluids would not yet have formed to any significant extent. I actually do not think it would have been particularly unpleasant. And that is part of dignity too, moving people as quickly as possible and preventing those processes from developing.” Military Physician

“In my view, the aspect of “indignity through delay” needs to be considered when choosing solutions. In sunlight, remains can deteriorate quickly. In that sense, speed in the transportation chain is itself a form of dignity.” QQP 36

Military Chaplain 1, drawing on previous experience with human remains, also emphasized the importance of decomposition processes:

“Several times I found myself thinking about the people carrying the bodies and how they were being handled. It also raised questions. For those doing this work, is it really sustainable to stand with your face close to the bags while tightening straps? How can people maintain a manageable distance over time? And what will these vehicles smell like? As clergy we have been close to bodies that have been dead for a long time, but usually that is one body in a coffin. If there are 14 bodies that have been lying for several days in the sun, then transported by truck and left standing in the heat during rest periods, I think this is something that needs to be carefully evaluated and learned from.”

From the medical perspective, dignity was closely linked to transporting the fallen as quickly as possible, taking decomposition processes into account. This was regarded as more dignified both for the deceased and for those responsible for caring for them.

### The logistical perspective

A logistical perspective also emerged concerning how the fallen were transported, for example in trucks. Since the Swedish Armed Forces currently lacks a dedicated mortuary affairs unit, the mission was carried out by temporary composite units assembled for the exercise. Transporting 600 bodies represented a major logistical undertaking. With limited preparation time, logistics personnel had to determine methods that balanced dignity with maintaining transport capacity. Depending on the vehicles and units involved, these solutions varied and were perceived differently by participants, a finding also noted in Observation Protocols 18, 26, and 83.

“I also reflected on our method of transporting the dead. Fourteen fallen personnel was probably the maximum number that could be transported with dignity. I felt that another unit’s method fell short. It seemed as if their plan was simply to load them in as quickly and easily as possible, which I think was wrong.” QQP 14

From a logistical perspective, there was a lack of standardized dignified solutions for transporting the fallen, particularly regarding trucks and other vehicles. This led to different interpretations of how the principle of dignity should be implemented in practice.

## Threats to human dignity

### The entire logistical chain must function with dignity

The exercise generated numerous observations regarding factors that threatened the experience of dignity. One recurring theme concerned the need for the entire logistical chain, involving both the Swedish Armed Forces and civilian actors, to maintain a corresponding level of dignity. Otherwise, discrepancies in perceived dignity could arise during transfers between organizations or when receiving organizations lacked the capacity to manage the number of fallen personnel involved or to do so at the desired pace (also noted in Observation Protocols 3, 26, 56). The result could be transport delays, bottlenecks, frustration, and a perception of indignity.

“The Church of Sweden’s preparedness and ability to receive the bodies. There was a lot of waiting during unloading because of a lack of stretchers and unloading space. Sometimes we could only unload one vehicle at a time.” QQP 4

“The Church of Sweden’s capacity to receive the fallen was limited, which ultimately meant they could not handle all the dead. Because of this, the final transports were not unloaded as dignifiedly as they should have been.” QQP 7

Participants also described experiences of having to carry bodies long distances across rocky terrain and place them in various locations at different stages before they were ultimately transported to the Church of Sweden. Teams of four, and sometimes only two, carried the body bags by hand over these distances. This was time consuming, physically demanding, and not always easy to accomplish in a dignified manner, as the heavy bags tended to collapse inward, heels occasionally pressed against the bags, and the bags sometimes dragged along the ground (also noted in Observation Protocols 10, 20, 23, 24, and 44). Stretchers could have substantially facilitated this process but were either unavailable or not used (also noted in Observation Protocols 44, 39, 24, 19, and 3).

“In my opinion, the fallen should have been carried between loading points on stretchers. That would have made carrying easier and helped maintain dignity by ensuring that bodies were not dragged along the ground.” QQP 16

In addition, the body bags did not always hold up during handling (also noted in Observation Protocols 44 and 19).

“The body bags broke very easily. I experienced this as undignified. The seams split when lifting them. Real bodies weigh more than the mannequins used in the exercise, which means the bags would likely fail even more often in a real situation.” QQP 87

### The time factor

A clear theme was that stamina and endurance, and thus the ability to maintain dignity, declined over time. This was observed in Observation Protocols, interviews, and qualitative responses.

“By the end of the day, I think people wanted to finish the task and unfortunately dignity declined somewhat.” QQP 20

“As time passed, the atmosphere became more relaxed, which probably would not have happened in a real situation.” QQP 63

“The scale and duration of the exercise meant that dignity and respect gradually decreased. People became more focused on efficiency, which led to more of a “use and discard” mentality.” QQP 70

“At the beginning we carried the bags four at a time, which the chaplain considered dignified. Later we carried them two at a time to be more efficient. At first I felt that we were carrying individuals. Later we were carrying bags. By the end everything became faster and less careful. What mattered most was getting it done.” QQP 77

An important observation concerning the relationship between time, fatigue, and dignity emerged from Military Chaplain 2, who participated in the exercise rather than serving as an observer. Remaining continuously present alongside the service members throughout the exercise, the chaplain reflected on how this sustained presence, together with visible demonstrations of respect for the fallen, appeared to reinforce respectful conduct and help preserve dignity throughout the exercise.

“Before dinner everyone was exhausted, myself included. People were starting to wear down. I heard someone tell another person, “carry the bag with dignity, do not drop it.” I chose not to intervene in that way. I just heard it being said. But I could see that some people were dragging their feet and beginning to tire. So I chose to walk alongside them or stand visibly near the dead and show my respect. I noticed that it became quieter around the area, even though people kept working.” Military Chaplain 2 (this event is described in a similar manner in Observation Protocol 23)

In Observation Protocols, for example 17, 23, 39, 41, 83, 84, and 85, as well as in additional interviews, for example with Military Chaplain 1, it was also possible to discern a decline in dignity over time. This was expressed through participants eating very close to the body bags, water and refreshment stations being placed only a few meters from the body bag area, people beginning to take shortcuts across collection areas, litter appearing, and the atmosphere at times becoming more relaxed and occasionally humorous.

“During transport, someone stepped on the body bags and joked about the fact that in reality they would have contained my comrades. At the time it did not seem like a big deal, but looking back it was clearly inappropriate.” QQP 87

Other instances of humorous behavior during the exercise were also noted in Observation Protocols 17, 23, 42, and 83. Increased stress toward the end of the exercise further contributed to the perception that dignity declined and was, to some extent, lost.

### Documentation

Documentation was a central component of the exercise. The fallen were assigned identities created by the Swedish Tax Agency, and these identities had to be processed from pronouncement of death through the involvement of relevant authorities and the Church of Sweden for emergency burials. Limited previous experience, the absence of established routines, and varying levels of training among personnel created challenges that affected the ability to care for the fallen in a dignified and respectful manner.

“These challenges concerned issues ranging from pronouncement of death and identity tags to death reports, personal belongings, and procedures governing how long bodies could remain in body bags.” Military Physician

Most of the uncertainty concerned administrative procedures. Everything from filling out death reports and knowing where they should go, to identity tags and what should be done with them. I do not think anyone even removed the tags, so there were many questions like that.

The limitations of the body bags, as noted above, could also have significant consequences.

“The tags attached to the bags often fell off because they were not secured properly. That creates the risk of losing track of who is in which bag, which would be extremely problematic.” QQP 43

Many of these observations concerning deficiencies in procedures and equipment were also documented in Observation Protocols 3, 5, 7, 10, 26, 32, 49, and 56. Experiences of not knowing how to proceed, together with equipment and materials that did not function as intended, contributed to feelings of indignity in the handling of the dead. Education and training, along with greater standardization and the development of clear procedures, were the most common recommendations for addressing the dignity related challenges that participants experienced in relation to documentation.

## Discussion

Several important insights emerge from this study and merit further elaboration.

### Experiencing the meaning and burden of mortuary affairs

The first relates to the extent to which participants’ experiences during this exercise, albeit in a substantially reduced form, mirror findings reported in the mortuary affairs literature. Participants expressed the importance of mortuary affairs and of ensuring that fallen service members are returned home to their resting place (13). Previous research has shown that such work can foster a strong sense of pride and purpose (13), a finding that was also evident in the present exercise. At the same time, participants demonstrated a deeper understanding of the emotional burden associated with these tasks, while also describing experiences of greater maturity and personal growth, closely mirroring findings among mortuary affairs personnel reported in previous research (13). Even humor was evident among the participants, mirroring findings from studies of mortuary affairs personnel, who frequently employ humor as a coping mechanism when processing and discussing their experiences (13). However, for outsiders or those unacquainted with this line of work, such humor may be perceived as irrelevant, callous, or disrespectful, despite serving important psychological and social functions (32).

What an exercise cannot fully reproduce, however, is the psychological and emotional toll associated with mortuary affairs under operational conditions. Existing research has documented the psychological burden associated with this work, including symptoms of PTSD (17–19). In particular, studies have found that inexperienced personnel and non-volunteers exhibit higher levels of intrusion and avoidance symptoms (17, 18). These findings suggest that, in an actual operational context, it may be advantageous to assign such responsibilities to experienced, well trained, and voluntary personnel within a dedicated professionalized mortuary affairs unit. Ideally, such personnel would also have limited personal connections to the deceased. This may help reduce the psychological costs associated with these duties.

These considerations are further complicated by the character of contemporary warfare. Experiences from Ukraine demonstrate that casualty recovery and mortuary affairs are far from straightforward. Ashbridge et al. (6) highlight the challenges posed by hostile environmental conditions and bodily decomposition for the long-term management of war fatalities and the identification of the dead. Such circumstances make it particularly difficult to maintain the dignity and respect that all parties seek to uphold, while also underscoring that mortuary affairs activities may continue long after the cessation of hostilities. Personnel involved in recovery and mortuary affairs operations may therefore be exposed to severely decomposed remains and extensive traumatic injuries, experiences that can be highly demanding both emotionally and psychologically (6, 13).

### Expanding the mental health perspective through moral injury

Much of the mortuary affairs literature cited in this article was published before moral injury became established as a distinct clinical construct (33–35). Consequently, the psychological consequences of mortuary affairs have primarily been conceptualized through the lens of traumatic stress, with particular emphasis on PTSD symptoms and related psychological outcomes. Although this perspective has generated important knowledge, it may not fully capture the moral and existential challenges inherent in caring for war dead. Incorporating a moral injury perspective may therefore provide a more comprehensive understanding of the psychological consequences of mortuary affairs.

One of the clearest morally salient issues reflected in the present study concerned participants’ reflections on situations in which wartime circumstances prevented the timely recovery, identification, and evacuation of fallen service members. Such failures may be experienced not merely as operational shortcomings but as moral transgressions, violating the deeply held obligation to honor and care for the dead (33). Unlike combat-related moral injury, personnel engaged in mortuary affairs may be exposed to potentially morally injurious events (PMIEs) rooted not primarily in acts of violence, but in perceived failures to recover, identify, care for, and honor the dead.

Other morally challenging situations may arise when personnel are required to recover, identify, and temporarily bury large numbers of fallen service members following mass-casualty events. Such experiences may evoke profound moral questions, including: Why did so many people die? Whose decisions placed these service members in harm’s way? Could these deaths have been prevented? Were mistakes made? These questions extend beyond traumatic exposure and concern the moral meaning attached to death, responsibility, military leadership, and institutional accountability.

As Litz et al. (33) argued, PMIEs may arise whenever service members die, kill, fail to prevent death, witness unnecessary death or suffering, desecrate human remains, or encounter remains that have been desecrated. Personnel engaged in mortuary affairs may therefore be repeatedly exposed to situations with considerable potential for moral injury.

Future research should complement assessments of PTSD and other trauma-related outcomes with qualitative exploration of morally distressing experiences, as well as quantitative assessment using validated measures of PMIE exposure and moral injury (36). Incorporating such assessments into after-action reviews following mass-casualty events could facilitate the early identification of moral distress, strengthen organizational learning and preparedness, and promote adaptive posttraumatic growth among military personnel involved in mortuary affairs.

### Negotiating dignity in mortuary affairs operations

A third important insight concerns the concepts of dignity and respect. These are culturally and contextually situated concepts whose meanings are neither fixed nor universally shared. Dignity may be understood from multiple perspectives, including ecclesiastical, medical, and logistical perspectives. Each of these perspectives is embedded within institutional frameworks characterized by distinct values, meanings, and practices (37). Although this exercise will likely contribute to the development of methods, routines, and standardization, each mortuary affairs operation conducted during conflict or war will inevitably require balancing and negotiating these different understandings of dignity and respect against the specific conditions of the operation. Such conditions may include human, technological, and tactical considerations, including threats from drones, sniper fire, and adverse weather conditions.

**Figure 1:**
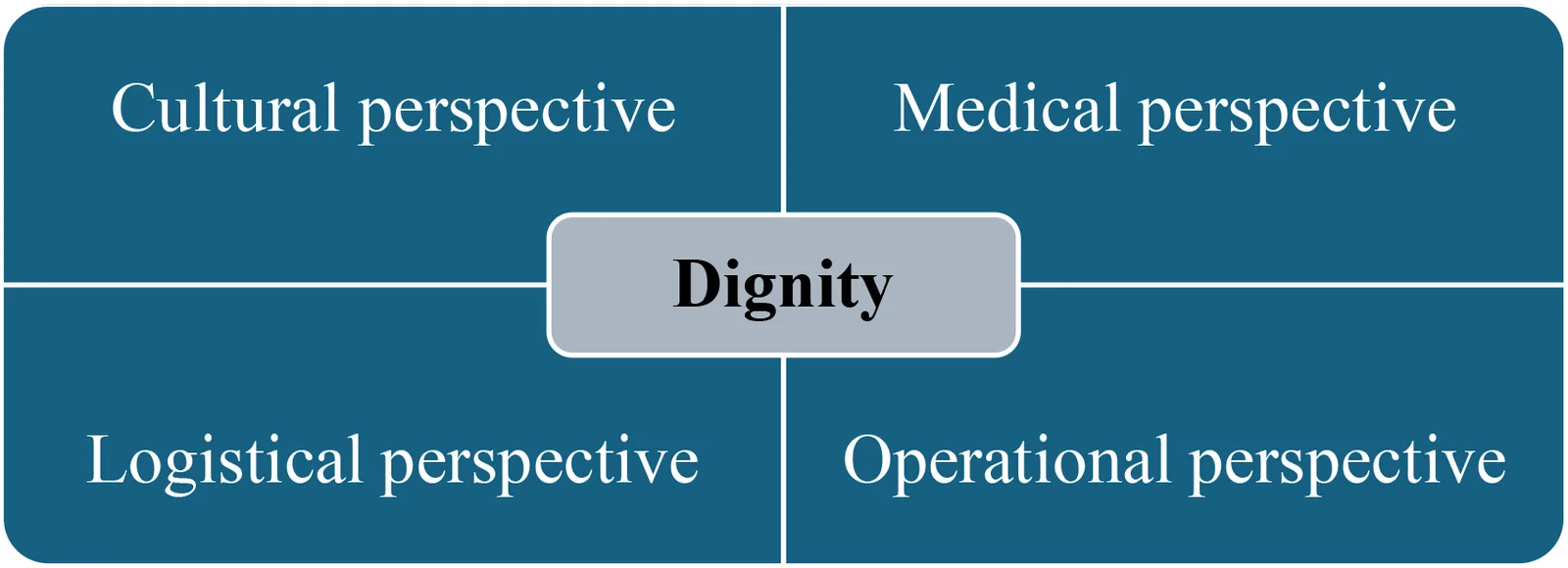
Negotiating dignity in mortuary affairs.

As illustrated in **Figure 1**, dignity may therefore be conceptualized as a negotiated construct that emerges through the balancing of at least these four perspectives, with their relative importance varying across mortuary affairs operations.

Complete standardization of mortuary affairs operations with respect to dignity is unlikely to be attainable in full-scale war (6). Likewise, as Dias (29) argues, a single, universally accepted definition of human dignity is difficult to achieve because conceptions of dignity are shaped by societal complexity, cultural traditions, and differing practices surrounding death. The four perspectives identified in the present study should therefore be understood as complementary analytical lenses for planning and conducting mortuary affairs operations, rather than as prescriptive standards. Although standardized procedures remain essential for ensuring respectful and dignified handling of the dead, the relative importance and practical application of these perspectives will inevitably vary according to the operational, cultural, and situational context.

### Sustaining dignity during prolonged operations

From an operational perspective, the temporal dimension is also important. The findings clearly indicate that maintaining dignity became increasingly difficult over time, already after approximately four hours of activity. Although the experience might have differed under actual operational conditions, additional factors absent from the exercise would likely further complicate the situation, including exposure to decomposition and the handling of the remains of real individuals. If dignity is to be maintained during prolonged operations, mortuary affairs activities will likely need to be organized differently to ensure that personnel can sustain dignified standards of care over extended periods.

### The ministry of presence as a reminder of dignity

The findings also suggest that the presence of military chaplains, often described in the chaplaincy literature as a ministry of presence (38, 39), may help preserve dignity when it is at risk of being compromised. In the Swedish context, military chaplains are ordained priests in the Church of Sweden, and many service members will have encountered them previously in connection with death, funerals, and bereavement in civilian life (25). Their presence may therefore serve as a reminder of the social, ethical, and moral norms associated with death, reinforcing expectations of respectful and dignified treatment of the fallen.

In this sense, military chaplains may serve a role that extends beyond providing spiritual care, emotional support, and comfort. Without necessarily directing behavior, their presence may help sustain ethical awareness during situations in which operational demands and psychological strain risk diminishing attention to dignity. Thus, the ministry of presence (38, 39) may not only support the psychological well-being of personnel but also reinforce the dignified handling of the dead by reminding service members of the moral values that underpin military service and the respectful treatment of fallen service members.

### The moral significance of mortuary affairs

A sixth important finding concerns the moral significance of a well functioning mortuary affairs system. Existing research emphasizes not only practical and organizational dimensions but also the profound moral importance of dignity, respect, and appropriate conduct toward fallen service members (12–16). Such experiences may have implications for both individual wellbeing and military effectiveness, including warfighting capability (10).

For armed forces seeking to uphold their moral obligations during war while maintaining operational effectiveness, the ability to care for fallen service members in ways perceived as appropriate by both surviving personnel and bereaved families is of considerable importance. A well functioning mortuary affairs capability is therefore not merely an organizational necessity but also a moral imperative.

One challenge is that many armed forces have not yet fully adapted from an era characterized by relatively low casualty rates (9), associated with decades of small scale and professionalized military operations such as those conducted during the so called “Global War on Terror” (7, 8), to the realities of large scale warfare requiring the capacity to manage substantial numbers of war dead (6, 10). Developing a mortuary affairs capability capable of functioning under conditions of large-scale war may therefore be viewed as strategically important from both a moral and an operational perspective. If such systems fail, the concept of moral injury (40, 41) offers a useful framework for understanding the potential consequences for service members who perceive themselves as betrayed in relation to what is morally right in high-risk situations. Such experiences may contribute to feelings of disillusionment, bitterness, cynicism, and frustration.

### Training for dignified mortuary affairs

The final finding highlights the importance of exercises such as the one examined in this study. It is difficult to overstate the importance of training casualty recovery, triage, mortuary affairs, and emergency burials at all organizational levels, from individual service members and platoons to companies, battalions, higher tactical and strategic headquarters, and relevant civilian actors involved in the broader process. Such training is essential if these activities are to function effectively under operational conditions. Beyond enhancing operational preparedness, it enables personnel to develop a deeper understanding of death in war, the realities of caring for the fallen, and the emotional, practical, and moral challenges such responsibilities may entail.

Moreover, dignity must be maintained throughout the entire chain of mortuary affairs activities. This requires all actors involved, military as well as civilian, to uphold a corresponding standard of dignity and respect in their interactions with the dead. Otherwise, failures at any stage of the process may compromise the perceived dignity of the operation as a whole.

As many participants observed, death is an inevitable feature of war, yet these tasks are rarely exercised. Mortuary affairs should therefore be integrated into regular military training and practiced by all relevant personnel. If mortuary affairs are to be conducted with dignity and respect during wartime, they must be exercised frequently and extensively during peacetime.

This is not solely a military concern but a societal one. The ability to manage war dead in a dignified and satisfactory manner during peacetime is essential for maintaining such capability during war. A society’s response to its war dead reflects the values, norms, and emotions it seeks to associate with death in war (14, 16). Ultimately, this capability also reflects the moral intentions and norms codified in international conventions (21) and national legislation, including the Swedish Burial Act (22).

## Concluding remarks

In light of the findings of this study, including participants’ experiences of dignity and respect during the exercise, careful consideration should be given to the relative merits of *ad hoc* mortuary affairs arrangements versus a dedicated mortuary affairs unit.

From psychological, operational, and dignity related perspectives, a dedicated, well trained, and well equipped unit appears preferable. A unit trained in relevant procedures, behaviors, and rituals, and equipped with specialized vehicles and equipment, is likely to be better positioned to conduct mortuary affairs operations in a professional, consistent, and dignified manner.

From a moral injury perspective, such a specialized unit may also be better equipped to prepare for, recognize, and navigate the moral challenges that can arise before, during, and after mortuary affairs operations.

Such a unit would also be able to develop expertise through repeated operations and the continuous exchange of lessons learned with relevant international partners, including actors involved in the war in Ukraine.

At the same time, all military personnel require a basic understanding of mortuary affairs in order to manage the deceased appropriately until a specialized unit can assume responsibility. Training and exercises in this area may also provide valuable educational and professional insights.

Finally, warfare is inherently unpredictable. Even where a specialized mortuary affairs capability exists, temporary or improvised solutions will sometimes be necessary. Such adaptability should therefore complement, rather than replace, a dedicated mortuary affairs capability.

### Limitations and future research

All research has its limitations. Although the qualitative questionnaires employed in this study provided rich insights into a substantial portion of the participants’ subjective experiences, the design did not allow for follow up questions or further probing of individual responses. As a result, the interpretation of the data was necessarily constrained by the information provided, requiring a cautious and conservative approach to analysis.

No service members at the soldier or seaman level were interviewed; instead, the interview sample consisted of professional experts and one commanding officer. Consequently, the study provides only limited in-depth insight into the experiences of service members at the soldier or seaman level, whose perspectives could not be fully captured through the qualitative questionnaire alone.

Approximately one third of all exercise participants completed the qualitative questionnaire. Most responses were provided by personnel directly involved in the core activities of the exercise, such as the handling and transportation of bodies. As a result, perspectives from personnel in more peripheral, yet potentially important, support functions may be underrepresented.

Finally, future exercises could include a substantially larger number of simulated fallen enemy soldiers to explore whether differences emerge in the handling of Swedish and enemy war dead. This would also provide an opportunity to examine important ethical and moral challenges, including ceremonial practices, both within the military context and at sites designated for the temporary burial of the dead.

Future research should further examine mortuary affairs in the context of the war in Ukraine and identify lessons that may inform both research and practice in this field.

## Data Availability

The original contributions presented in the study are included in the article/**Supplementary Material**. Further inquiries can be directed to the corresponding author.
