# Supplementary material for: Experiences of caring for large numbers of fallen service members in a realistic large-scale mortuary affairs and emergency burial exercise: maintaining dignity and respect during Aurora 26
Source: Front Psychiatry. 2026 Aug 28;17:1907283. doi: 10.3389/fpsyt.2026.1907283 (PMC13564260; doi:10.3389/fpsyt.2026.1907283)
Supplement: Supplementary file 1 [file DataSheet1.pdf]

The study has been approved by the Swedish Ethical Review Authority (Reference 2026-01258-01).
Principal Investigator Jan Grimell
Department of Sociology
Umeå University

Observation protocol for an observational study of mortuary affairs (English version)

1. Basic Information

Date: __________________ Time (start-end): __________________

Observation area/position: ____

Observer’s name: ____

2. Purpose & focus

- To study how dignity and respect are maintained and upheld in mortuary affairs operations.
- To examine how moral factors (human attributes such as willpower, courage, resilience, esprit de corps, leadership, education, exercises, training, and ethics) are influenced by participation in mortuary affairs operations.
- To study the Swedish Armed Forces’ core values.
- To observe and analyze behaviors and reactions associated with mortuary affairs operations.

The bullet points above summarize the areas that you, as an observer, should focus your observations on. Some aspects may not be visible or manifested during the observation, while others may become particularly evident. Likewise, concepts such as dignity within the context of Mortuary Affairs are not always easy to define precisely, as their meaning may vary depending on the situation and circumstances.

3. Instructions for documenting observations

During your observation session, record what you see and hear in a neutral and objective manner. Avoid interpretations and evaluative language, except in the comments section, where a more interpretive tone may be appropriate. Take notes continuously throughout the entire observation period. The purpose of the notes is not to document a single dramatic event, but rather to develop an ongoing understanding of how the entire process unfolds in the location where you are observing. Avoid recording identifying details that could make it possible to identify the individuals being observed. Your observations should be documented according to the following structure and will serve as the basis for the researcher’s analysis:

- Time (or a time range, e.g., “Between 13:00 and 15:00” to describe a longer period)
- What happened
- Effect/Outcome
- Comments

Note: A new observation protocol should be completed for each observation session.

The study has been approved by the Swedish Ethical Review Authority (Reference 2026-01258-01).
Principal Investigator Jan Grimell
Department of Sociology
Umeå University

Documentation of observations

The study has been approved by the Swedish Ethical Review Authority (Reference 2026-01258-01).

Principal Investigator Jan Grimell
Department of Sociology
Umeå University

The study has been approved by the Swedish Ethical Review Authority (Reference 2026-01258-01).
Principal Investigator Jan Grimell
Department of Sociology
Umeå University

The study has been approved by the Swedish Ethical Review Authority (Reference 2026-01258-01).
Principal Investigator Jan Grimell
Department of Sociology
Umeå University

The study has been approved by the Swedish Ethical Review Authority (Reference 2026-01258-01).
Principal Investigator Jan Grimell
Department of Sociology
Umeå University

The study has been approved by the Swedish Ethical Review Authority (Reference 2026-01258-01).

Principal Investigator Jan Grimell
Department of Sociology
Umeå University

The study has been approved by the Swedish Ethical Review Authority (Reference 2026-01258-01).

Principal Investigator Jan Grimell
Department of Sociology
Umeå University

The study has been approved by the Swedish Ethical Review Authority (Reference 2026-01258-01).
Principal Investigator Jan Grimell
Department of Sociology
Umeå University

The study has been approved by the Swedish Ethical Review Authority (Reference 2026-01258-01).
Principal Investigator Jan Grimell
Department of Sociology
Umeå University

The study has been approved by the Swedish Ethical Review Authority (Reference 2026-01258-01).
Principal Investigator Jan Grimell
Department of Sociology
Umeå University

The study has been approved by the Swedish Ethical Review Authority (Reference 2026-01258-01).
Principal Investigator Jan Grimell
Department of Sociology
Umeå University

The study has been approved by the Swedish Ethical Review Authority (Reference 2026-01258-01).
Principal Investigator Jan Grimell
Department of Sociology
Umeå University

4. Overall impressions and assessment

4.1 Dignity and respect

How were dignity and respect maintained and upheld: 1 ☐ 2 ☐ 3 ☐ 4 ☐ 5 ☐ (1 = not at all, 5 = very well)

Any issues noted? ☐ No ☐ Yes: ____

4.2 Moral factors

To what extent were moral factors affected: 1 ☐ 2 ☐ 3 ☐ 4 ☐ 5 ☐ (1 = Not at all, 5 = To a great extent)

Comments (please specify): ____

4.3 Core values

Core Values

How well were the Swedish Armed Forces' core values upheld: 1 ☐ 2 ☐ 3 ☐ 4 ☐ 5 ☐ (1 = not at all, 5 = very well)

Comments: ____

4.4 Behaviors/reactions

To what extent were observable behaviors and reactions evident: 1 ☐ 2 ☐ 3 ☐ 4 ☐ 5 ☐ (1 = Not at all, 5 = To a great extent)

Comments: ____

5. Critical incidents

Mortuary affairs operations are, in themselves, demanding, emotionally challenging, and taxing activities when conducted in an operational environment. As a whole, such operations may be regarded as a critical incident. However, what is sought here is whether any specific events occurred during the observed activity in which dignity, respect, moral factors, core values, or observable behaviors and reactions were visibly affected by the mortuary affairs operation.

Critical incident #1
Time:

Description:

Consequence:

Cause:

Actions taken:

Critical incident #2

Time:

Description:

Consequence:

Cause:

Actions taken:

Critical incident #3

Time:

Description:

Consequence:

Cause:

Actions taken:

6. Summary

How would you summarize your observations in terms of dignity and respect, moral factors (human attributes such as willpower, courage, resilience, esprit de corps, leadership, education, exercises, training, and ethics), core values, as well as behaviors and reactions during Mortuary Affairs operations?

What worked well?

What could be improved to enhance Mortuary Affairs operations in an operational environment?

Additional reflections?

When you have completed the protocol: Please submit your observation protocol to the Principal Investigator, Jan Grimell, or place it in a temporarily designated collection box.

Appendix: Brief guide for observers

- Remain neutral and record, as concretely and simply as possible, what you see and hear.
- Record times to an extent that provides the researcher with a clear understanding of how the activity unfolded at the observation site.
- Choose an observation position that ideally allows you to both see and hear the activity.
- It is perfectly acceptable to move to new locations or positions during the observation. If you do so, make sure to note this in the protocol so that the researcher can follow your observations when reviewing the protocol.
- The purpose of the notes is not to document a single dramatic event, but rather to develop an ongoing understanding of how the entire process unfolded in the location where you were observing.
- Distinguish between observation and interpretation.
  Observation: "Person A places the body on the ground at 02:14."
  Interpretation: "He was stressed."
- If you are uncertain, record what you observed and mark it with a "?".
- Avoid recording identifying details that could make it possible to identify the individuals being observed. Individuals described in relation to an event should be de-identified.
- Submit your observation protocol at the end of the observation session (to Jan Grimell or to a temporarily designated collection box).
- Always begin a new protocol for each observation session. If the observation session is particularly long, use two protocols.
- Bring extra pens and a hard surface to write on.
- Have a strategy for adverse weather conditions so that you can still perform your duties as an observer, including maintaining adequate documentation throughout the session.
- Write clearly and legibly.

The following elements of the Swedish Armed Forces' core values are of particular interest:

- Demonstrating ethical and moral awareness when operating among and handling fallen personnel.
- Complying with applicable laws and conventions governing the dignified, respectful, and reverent handling of the deceased (both friendly and enemy dead).
- Showing care and support toward colleagues who may find the situation emotionally challenging.
