# Supplementary material for: Experiences of caring for large numbers of fallen service members in a realistic large-scale mortuary affairs and emergency burial exercise: maintaining dignity and respect during Aurora 26
Source: Front Psychiatry. 2026 Aug 28;17:1907283. doi: 10.3389/fpsyt.2026.1907283 (PMC13564260; doi:10.3389/fpsyt.2026.1907283)
Supplement: Supplementary file 2 [file DataSheet2.pdf]

UMEÅ UNIVERSITET

Information om forskningsprojektet *Krigsgravtjänst i en nutidskontext*

Jag vill fråga dig om du vill delta i forskningsprojektet *Krigsgravtjänst i en nutidskontext* och i det här dokumentet får du information om projektet och vad det innebär att delta.

Bakgrund

Inom Försvarsmakten benämns arbetet med stupad personal krigsgravtjänst. Den omfattar hela omhändertagandekedjan fram till dess att den stupade, om möjligt, överlämnas till den huvudman som ansvarar för begravningsverksamheten i Sverige. Att krigsgravtjänsten fungerar väl är av betydelse för de moraliska faktorerna, det vill säga mänskliga egenskaper såsom vilja, mod, tålighet, anda, ledarskap, utbildning och etik, inom ett militärt förband och i förlängningen även förbandets krigsföringsförmåga. Krigsgravtjänst är emellertid i stor utsträckning en oövad praktik i en svensk nutidskontext, särskilt i relation till de omfattande dödstal som kriget i Ukraina tydligt visar kan uppstå i dagens krigföring. Det saknas i dag nutidsforskning om detta i en svensk militär kontext.

Vad är det för projekt och varför din medverkan är viktig

Syftet med forskningsprojektet är att få en fördjupad förståelse för hur krigsgravtjänsten i en nutida kontext påverkar moraliska faktorer hos militär personal och de etiska dimensionerna i omhändertagandekedjan, samt hur detta i förlängningen kan påverka krigföringsförmågan.

Din erfarenhet efter genomförd övning i krigsgravtjänst är unik. Genom att du efter övningen besvarar frågorna i den kvalitativa enkäten eller deltar i intervjuerna får jag en möjlighet att undersöka dina erfarenheter och upplevelser av krigsgravtjänsten.

Hur går studien till?

Studien är upplagd med en kombination av insamlingsmetoder. Dels genomförs en kvalitativ enkät med merparten av deltagarna, dels genomförs intervjuer med ett mindre, avgränsat urval av deltagare i särskilda befattningar.

<u>Kvalitativ enkät</u>

I den kvalitativa enkäten finns ett antal frågor som du får fylla i för hand. Enkäten är helt anonym och kommer inte att kunna spåras tillbaka till dig. Enkäten tar ungefär 30 minuter att fylla i. När du är färdig samlas enkäten in av ansvarig person på plats och skickas till forskaren.

Innan du fyller i enkäten får du skriva under ett samtyckesformulär, det är viktigt att du samtycker till att delta.

Ingen obehörig kommer att få ta del av enkätsvaren som kommer att förvaras inlåst av forskaren.

<u>Intervju</u>

För dig som intervjuas tar intervjun cirka 45 minuter och genomförs på en lämplig, avskild plats i anslutning till övningsområdet. Forskaren kommer att ställa öppna frågor som relaterar till din befattning och den genomförda övningen, och du besvarar frågorna fritt.

Innan intervjun får du skriva under ett samtyckesformulär. Ingen obehörig kommer att ta del av intervjusvaren. Intervjuer och utskrifter kommer att kodas och förvaras inlåsta.

Möjliga följder och risker med att delta i studien

Att besvara den kvalitativa enkäten och delta i intervju bedöms inte innebära någon risk för dig som deltar. Deltagandet är frivilligt. I enkäten lämnar du dina svar anonymt och den går inte att spåra tillbaka till dig.

Under intervjun kommer jag att ställa frågor om dina upplevelser och erfarenheter i din befattning under övningen. Intervjun kommer att hanteras på ett sådant sätt att din integritet skyddas.

Min avsikt är att redovisa resultaten på ett sätt som så långt som möjligt förhindrar bakåtspårning.

Vad händer med mina uppgifter?

Den kvalitativa enkäten kommer att förvaras inlåst i skåp och är endast tillgänglig för behöriga. Enkäten kan inte spåras till dig om du följer instruktionerna, inte ens jag vet vilken enkät som är just din.

Intervjuer kommer att spelas in och översättas till skriven text. Allt material förvaras inlåst i skåp eller på lösenordsskyddad dator under transport och bearbetning och är endast tillgängligt för behöriga. Ljudfilerna raderas efter överföring men sparas inlåst på extern hårddisk tills projektet avslutats. I det transkriberade materialet kommer alla namn att ersättas med ett ID-nummer, för att minska risken för bakåtspårning. Allt förvaras inlåst i olika arkivskåp. Efter avslutat projekt kommer allt material att raderas.

Dina svar och dina resultat kommer att behandlas så att inte obehöriga kan ta del av dem.

Hur får jag information om resultatet av studien?

Information om projektet och de publikationer som görs kommer att finnas tillgängliga på hemsidan för sociologiska institutionen på Umeå universitet under mitt namn.

Frivilligt att delta

Det är frivilligt att delta i projektet. Om du väljer att delta kan du när som helst under projektets gång dra tillbaka din medverkan i projektet utan att ge något skäl och utan att det får några konsekvenser för dig.

Studien är etikprövad

Den här studien är etikprövad och godkänd av Etikprövningsmyndigheten och har diarienummer 2026-01258-01.

Så behandlar Umeå universitet dina personuppgifter i forskning

I den här studien inhämtas personuppgifter om dig via kvalitativ enkät och intervju. De personuppgifter som inhämtas och sparas är:

- Sociodemografiska data: kön, arbetsplats (Försvarsmakten), yrke, år i yrket
- Informerat samtycke (inlåst i arkivskåp)

Om du väljer att delta i ett forskningsprojekt där uppgifter om dig kommer att finnas gäller EU:s dataskyddsförordning 2016/679 (GDPR.) Här får du information om hur Umeå universitet behandlar dina personuppgifter i ett forskningsprojekt och vilka rättigheter du har enligt EU:s dataskyddsförordning. Det är Umeå universitet som är personuppgiftsansvarig för de person-

uppgifter som behandlas i forskningsprojektet. Umeå universitet kommer endast behandla personuppgifter som är nödvändiga för att uppnå ändamålet med forskningen. Umeå universitet har rätt att behandla personuppgifter om behandlingen är nödvändig för att utföra en uppgift av allmänt intresse (EU:s dataskyddsförordning, artikel 6.1 e). Forskning är ett sådant allmänt intresse. Uppgifterna kan också komma att behandlas för andra ändamål som ryms inom Umeå universitets uppdrag, till exempel skyldigheter som följer av lag (EU:s dataskyddsförordning, artikel 6.1 c).

Efter forskningen avslutas kommer Umeå universitet arkivera personuppgifter enligt bestämmelser i arkivlagen och universitetets dokumenthanteringsplaner. Arkivering innebär att personuppgifter sparas under en viss tid, vanligtvis i 10 år men i vissa fall längre. Umeå universitet omfattas av den så kallade offentlighetsprincipen, vilket innebär att alla som begär det har rätt att få ut kopior av allmänna handlingar som inte är sekretessbelagda. Dina personuppgifter kan därför även komma att lämnas ut vid en begäran om utlämnade av allmänna handlingar efter en noggrann sekretessbedömning. Uppgifterna kommer att behandlas så att inte obehöriga kan ta del av dem. Dina personuppgifter kommer att skyddas av lämpliga tekniska och organisatoriska åtgärder. Det innebär att dina personuppgifter kommer att hanteras och lagras i IT-system och tjänster som är godkända av Umeå universitet och skyddas genom lämpliga säkerhetsåtgärder. I det fall dina personuppgifter skickas till en mottagare utanför Umeå universitet sker det i enlighet med reglerna i EU:s dataskyddsförordning och i offentlighets- och sekretesslagen. Du kan kontakta den forskare som är ansvarig för forskningsprojektet om du vill ha mer information om hur dina personuppgifter hanteras och skyddas. Enligt EU:s dataskyddsförordning har du bland annat rätt att begära:

- tillgång till dina personuppgifter (så kallat registerutdrag)
- att få dina personuppgifter rättade
- att få dina personuppgifter raderade
- behandlingen av dina personuppgifter begränsas.

Det finns vissa undantag från dessa rättigheter. Umeå universitet kan till exempel i vissa fall inte radera personuppgifter på grund av regler rörande arkivering. Om du vill åberopa någon av dessa rättigheter ska du ta kontakt med dataskyddsombudet vid Umeå universitet (pulo@umu.se). Mer information om universitetets behandling av personuppgifter finns också på umu.se/gdpr. För att dataskyddsombudet ska kunna hjälpa dig behöver du tala om vilket forskningsprojekt som du har deltagit i. Om du är missnöjd med hur dina personuppgifter behandlas har du rätt att klaga hos Integritetsskyddsmyndigheten. Information om detta finns på myndighetens webbplats (imy.se).

Forskningshuvudman

Sociologiska institutionen på Umeå universitet är forskningshuvudman. Det innebär bland annat att institutionen är ansvarig för att forskningen sker i enlighet med villkor som har meddelats i samband med etikgodkännandet.

Ansvarig för studien

Jan Grimell, universitetslektor och docent, sociologiska institutionen, Umeå universitet.
E-mail: jan.grimell@umu.se
Mobil för sms: 0702235383

Mer information

Har du frågor eller funderingar ta kontakt med Jan Grimell.

UMEÅ UNIVERSITET

Informerat samtyckesformulär för studien *Krigsgravtjänst i en nutidskontext*

Var vänlig att läs igenom texten och kryssa i rutorna nedan:

Jag har fått muntlig och skriftlig information om studien och har haft möjlighet att ställa frågor. Jag får behålla den skriftliga informationen. ☐

Jag samtycker till att delta i den aktuella studien. ☐

Jag samtycker till att uppgifter om mig behandlas på det sätt som beskrivs i forskningspersonsinformationen. ☐

Jag förstår att jag när som helst kan avbryta mitt deltagande i studien utan att det blir några påföljder för mig som person. ☐

Ort och datum ……………………………………………………………….

Underskrift………………………………………………………………
