# Supplementary material for: Experiences of caring for large numbers of fallen service members in a realistic large-scale mortuary affairs and emergency burial exercise: maintaining dignity and respect during Aurora 26
Source: Front Psychiatry. 2026 Aug 28;17:1907283. doi: 10.3389/fpsyt.2026.1907283 (PMC13564260; doi:10.3389/fpsyt.2026.1907283)
Supplement: Supplementary file 3 [file DataSheet3.pdf]

Qualitative questionnaire: Mortuary affairs in a contemporary context (English version)

Background questions

- Position:
- Age:
- Previous international deployment(s):
- Gender:
- Length of service in the Swedish Armed Forces:

Please answer the questions below as descriptively and thoroughly as possible. Use the back of the paper if there is not enough space for your response.

Questions

1. What are your thoughts and reflections following this Mortuary Affairs exercise?

2. Based on your experiences during the exercise, how do you perceive that concepts such as dignity and respect were upheld?

3. Have any events or insights from the mortuary affairs exercise influenced how you think about the following aspects of your role in the Swedish Armed Forces in an operational (real-world) situation?

Will:

Motivation:

Courage:

Resilience:

Esprit de corps:

Leadership:

Ethics:

Education and training:

Core values:

4. From your perspective, were there any aspects of the mortuary affairs exercise that worked particularly well?

5. From your perspective, were there any aspects of the mortuary affairs exercise that did not work well and require further development?

6. How do you feel now, following the exercise, in terms of workload, stress, and emotional reactions?

7. Is there anything you would like to add that has not been addressed in this questionnaire?
