# Supplementary material for: Experiences of caring for large numbers of fallen service members in a realistic large-scale mortuary affairs and emergency burial exercise: maintaining dignity and respect during Aurora 26
Source: Front Psychiatry. 2026 Aug 28;17:1907283. doi: 10.3389/fpsyt.2026.1907283 (PMC13564260; doi:10.3389/fpsyt.2026.1907283)
Supplement: Supplementary file 4 [file DataSheet4.pdf]

Interview guide: Mortuary affairs exercise (English version)

Background questions

- How long have you served as a priest/military chaplain, military physician, or commander?
- What types of positions have you held as a priest/military chaplain, military physician, or commander?
- How long have you held your current position in the Swedish Armed Forces?
- Do you have any other military background or experience, such as overseas deployment or similar service?
- Age?
- Gender?

Interview questions

1. How would you describe your thoughts and reflections following this mortuary affairs exercise?
2. How do you experience serving as a military chaplain, military physician, or commander in a scenario involving a large number of fatalities?
3. What role and function did you assume during the exercise?
4. What expectations were placed upon you by others during the exercise?
5. How would you describe the cooperation and communication between yourself and others during the exercise?
6. To what extent do you believe that principles such as dignity, respect, and reverence for the deceased were upheld during the exercise?
7. How do you perceive that moral factors, such as will, courage, resilience, esprit de corps, leadership, education, training, and ethical values, were affected during the exercise?
8. Did you observe any particular behaviors or reactions, beyond what might normally be expected, in relation to the mortuary affairs activities?
9. What significance do you believe your background and expertise as a priest/chaplain, as well as ecclesiastical traditions and culturally embedded rituals, have for mortuary affairs operations?
10. Do you feel that you personally lack any education, training, or competencies necessary to perform this type of mortuary affairs duty effectively?
11. Do you believe that others, for example, commanders and seamen, lack any education, training, or competencies necessary to perform this type of mortuary affairs duty effectively?
12. Were there any aspects of the mortuary affairs operation that worked particularly well?
13. Were there any aspects that did not work well and require further development or improvement?
14. How do you feel now in terms of workload, stress, and emotional reactions?
15. Is there anything else you would like to add or return to before we conclude the interview?
